# Dr. Mihran K. Kassabian

**Published:** 1910-08

**Authors:** 


### OBITUARY

Dr. Mihran K. Kassabian, of Philadelphia, died at Jefferson Hospital in that city July 12, 1910, aged 42 years. His death was the result of *x*-ray burns received while experimenting with the rays, he being one of the most skilful and enthusiastic operators with that mysterious method of diagnosis and treatment. His life appears to have been a sacrifice on the altar of science. He was born in Asia Minor and came to the United States in 1894 to study medicine, serving in the hospital corps during the Spanish war. He graduated at the Medico-Chirurgical in 1898, and was immediately appointed instructor in electro-therapeutics and *x*-ray treatment in his alma mater. He was the author of several books relating to electrotherapeutics and radiography.

